# Statistical transformation of the population and social statistics system

**DOI:** 10.23889/ijpds.v8i2.2315

**Published:** 2023-09-14

**Authors:** Louisa Blackwell, Dom Webber

**Affiliations:** 1 Office for National Statistics, Fareham, United Kingdom

This presentation describes the proposed population and social statistics system, built around the Dynamic Population Model (DPM). The current population estimation system relies on the census. This provides granular data every 10 years, but the quality of population estimates declines between census years. The new system will deliver high quality population statistics, every year.

The DPM is a modelling approach which follows the cohort component method. It uses various data sources to measure population counts and components of population change, producing a coherent set of estimates. We use the DPM to produce admin-based population estimates for mid-year 2011 to 2022 for all local authorities in England and Wales. For international migration, we replace survey-based methods with administrative data. Rather than survey-based migration intentions, we estimate migration using observed activity.

The Coronavirus pandemic underlined the need for more timely population estimates. The DPM allows us to publish early, provisional estimates which are confirmed as the data feeds being used mature. In February 2023 we published research statistics for 2011-2022 mid-year population estimates, four months ahead of the date scheduled for the publication of the equivalent national statistics. We demonstrated the need to incorporate into the model a robust coverage adjustment method. Research is currently under way to optimise the coverage adjustment data and methods.

Our international migration estimates use different data sources and methods for each nationality grouping. We currently publish estimates on immigration, emigration and net migration for non-EU, EU and British nationals.

Transformation of the population and social statistics system will produce more timely and coherent estimates to better meet user needs. Flexible by design, our system will be able to reliably adapt to changes in social behaviour, changes in the data available for estimation and changes in users’ statistical needs.

